# Creation of the Youth Integration Project Framework: A Narrative Synthesis of the Youth Mental Health Integrated Care Literature

**DOI:** 10.5334/ijic.7730

**Published:** 2024-07-05

**Authors:** Michael Hodgins, Catherine McHugh, Valsamma Eapen, Gabrielle Georgiou, Jackie Curtis, Raghu Lingam

**Affiliations:** 1 Population Child Health Research Group, School of Clinical Medicine, University of New South Wales, Sydney, Australia; 2 Mindgardens Neuroscience Network, Sydney, Australia; 3 Discipline of Psychiatry, University of New South Wales, Sydney, Australia; 4 Academic Unit of Infant Child and Adolescent Psychiatry Services, South Western Sydney Local Health District, Australia

**Keywords:** youth mental health, integrated care, health services research

## Abstract

**Introduction:**

Integrated care has been posited as a potential solution to the global burden of youth mental health (YMH), but there is limited evidence on how best to design, staff, and evaluate different integrated care models. Our review aimed to consolidate the evidence on integrated models of mental healthcare for young people, to identify the core components of integration, and create a framework that can be used to analyse levels of YMH integration.

**Methods:**

We conducted a systematic review of literature across PubMed, SCOPUS, and PsycINFO databases and the grey literature We performed a narrative synthesis extracting core components of integrated YMH care.

**Results:**

Inductive themes from the literature described core components of integrated care. These themes were mapped into a novel framework combining the World Health Organisation health system building blocks and six intensity levels of integrated care to consider how best to implement and sustain integrated care within the YMH system.

**Discussion:**

The Youth Integration Project framework can form a basis for the development, implementation and evaluation of well-articulated models of youth integrated mental health pathways, assisting services identify what operational changes are needed to best implement and sustain integrated care.

## Introduction

Mental illness is the leading cause of disability-adjusted life years amongst people aged 0–24 years in high-income countries [1,2]. Globally, one in seven 10–19-year-olds experience a mental disorder, accounting for 13% of the global burden of disease in this age group [3]. In total, 75% of mental health disorders appear between adolescence and young adulthood, with increased severity over time [4]. These mental health disorders are associated with significant distress and long-term morbidity for young people (YP) and their families. Despite increased funding in high income countries to address mental health care needs for YP, existing models of mental health care are often inefficient, siloed, and have been associated with significant gaps in access to timely assessment and developmentally appropriate, evidence-based treatment [5,6]. Many YP and their families experience dissatisfaction and confusion in their interactions with the mental health system, describing a lack of person-centred care, where their preferences and needs are not addressed [7]. Further, existing service fragmentation poses challenges for YP accessing and navigating different siloed services, thereby limiting opportunities for cross service communication and collaboration [8]. Fragmentation leads to delays in seeking appropriate care, a lack of family and carer engagement, and YP are disproportionately affected by system fragmentation due to their need for parallel care of professionals from diverse disciplines and settings, especially at critical transitions [9].

The challenges to youth mental health (YMH) service delivery experienced globally have encouraged interest in whole-system reform and greater integration of care, posited to address the current mental health crisis [10]. The success of early psychosis models and services has spurred the development and implementation of broader integrated treatment models for YP and brought together mental health, physical health and social services [11]. Numerous specialised youth integrated care services that address physical and mental health issues, and in some instances also social issues, are in operation today, mainly in North America, Australia, and Europe. An integrated YMH care system has the potential to reach a greater number of YP, and their families with high-quality and evidence-based care and improve mental health outcomes [12].

The health systems-based perspective used by the WHO suggests that integrated care is achieved through alignment of all health system functions and effective change management. This includes organisational, functional, service, or clinical integration. Though this definition is comprehensive it lacks specificity on what constitutes integrated care, which is essential to inform how best to design, staff, and evaluate different models of integrated care for YMH. Work is needed to define the features of integrated YMH systems and identify the specific structural and systemic barriers and enablers to YMH service integration delivery. This work can lead to the co-production or adaptation of appropriate and feasible solutions to YMH service, system and care fragmentation for scale up. This has been insufficiently addressed in the emerging literature. To address this gap, our review aimed to consolidate the evidence on integrated models of mental healthcare for YP (defined as aged 12–25 years) and identify the core components of integration. Ultimately, we aim to contribute a blueprint of how best to implement and sustain integrated care within the YMH system.

## Theory and Methods

The systematic review was conducted in accordance with Preferred Reporting Items for Systematic Reviews and Meta-Analyses (PRISMA) guidelines [13]. Meta analysis of this review assessing the impact of integrated care on mental health outcomes has been published [14]. We undertook a review of peer-reviewed, English language research literature from January 2001–October 2021 using PubMed, SCOPUS, and PsycINFO databases and the grey literature. For the purposes of our narrative synthesis, we based our search on the research question: what are the core components of integration in YMH care? Studies that evaluated the effectiveness and implementation models of integrated mental health care for children and YP aged 12–25 years were included where they met the following eligibility criteria; 1) participants in the model had been diagnosed with at least one mental health condition, including Autism Spectrum Disorder (ASD) and Attention Deficit Hyperactive Disorder (ADHD), 2) studies were conducted in community-based settings. Studies were excluded where participants’ primary diagnoses were related to substance use disorders as a review of this literature already exists [15]. Additionally, literaure relating to models where mental health was only a small component, for example, literature relating to New Zealand Youth-One-Stop-Shops, which provide integrated health and social care to YP with complex needs, were excluded. This was determined by considering literature that described servies targeted towards YP with mental health related needs. While potentially providing insights into integration relating to YMH, this literature is much less specific to mental health system coinsiderations. Specific search terms are included in supplementary file 1. We also reviewed the websites of any named integrated care models identified in the research or grey literature for additional information on their service delivery model, and any information provided on the integrated approach the service had adopted.

### Identification of studies and extraction

**Figure 1:**
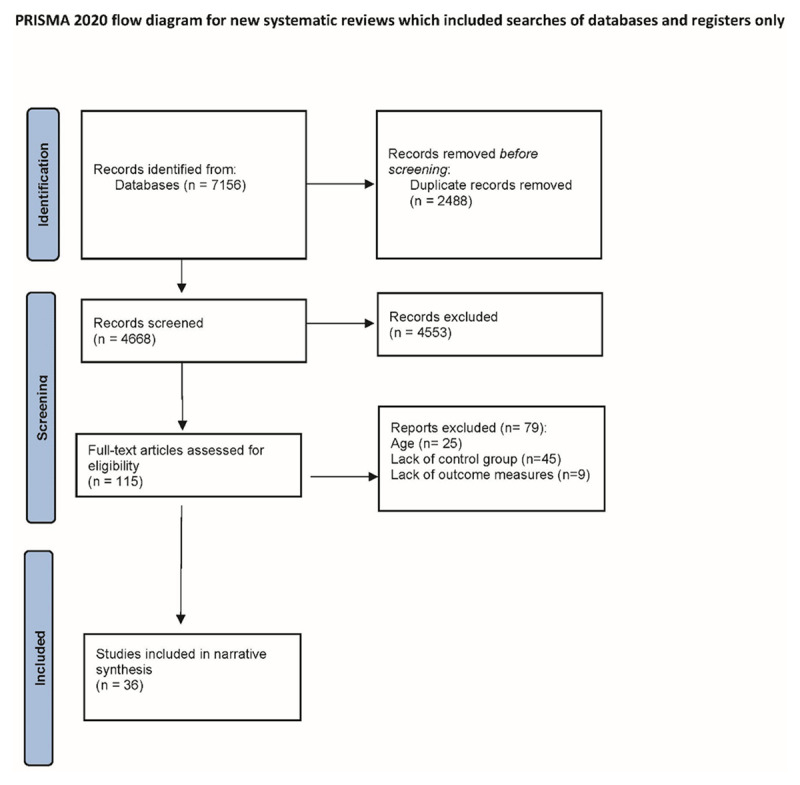
PRISMA flow chart.

Titles/abstracts were screened for relevance by one author (GG). The full-text of articles that were identified as potentially relevant after screening were reviewed by two authors to determine eligibility (GG, CM). Disagreements were resolved by consensus with the broader authroship team. See Figure 1 for PRISMA flow chart.

## Results

A total of 7,158 records were identified by searching the databases. After removing duplicates, 4,668 articles remained. After screening titles and abstracts, 115 full-text articles were assessed for eligibility. Out of these 115 articles, 79 articles were excluded. The reasons for exclusion were recorded, e.g. articles that describe models with no substantive elements of integrated care. **A total of 36 publications were retained for narrative synthesis**. We initially used a reflexive thematic analysis approach following that set out by Braun and Clarke by generating initial inductive themes describing core components of integrated care shared across the literature [16]. Our initial themes included multidisciplinary team-based care, communication and information systems, shared vision and common agenda, continuity of care and care coordination, and governance and funding. Noting the congruence between our intial inductive themes with the WHO building blocks of health services research, our themes were then deductively mapped against these building blocks. We have used this approach previously [17]. The WHO building blocks of health services research includes service delivery; workforce, health information systems; technology; finance; leadership; values allowing us to fully describe these core components of youth integrated mental health care. Table 1 shows a summary of the core components of integration taken from each included paper.

**Table 1:** Summary of the core components of integration taken from each included paper.


AUTHOR DATE	ARTICLE TYPE	COUNTRY	COMPONENTS OF INTEGRATED YMH CONSIDERED						

			SD	W	IS&C	P&T	F	V	LG&P

**Ådnanes and Steihaug, 2013**	Evaluation (implementation)	Norway	✔	✔	✔			✔	

**Asarnow et al 2015**	Literature review (meta analysis)	USA	✔	✔					

**Asarnow. et al 2005**	Evaluation (impact)	USA	✔	✔					

**Barbic et al 2022**	Evaluation (impact)	Canada	✔	✔				✔	

**Bartholomeusz 2022**	Literature review & evaluation (implementation)	Australia	✔	✔	✔	✔	✔	✔	✔

**Birchwood et al 2018**	Evaluation (impact & implementation)	UK	✔	✔	✔	✔	✔	✔	✔

**Callaly et al 2009**	Evaluation (implementation)	Australia	✔	✔				✔	✔

**Callaly et al 2011**	Literature review & evaluation (implementation)	Australia	✔	✔	✔	✔	✔	✔	✔

**Chiodo et al 2022**	Evaluation (implementation)	Canada	✔	✔		✔	✔	✔	✔

**Clarke et al 2005**	Evaluation (impact & implementation)	USA	✔	✔					

**De Voursney and Huang 2016**	Perspective	USA	✔	✔	✔	✔	✔	✔	✔

**Fusar-Poli 2019**	Literature review	UK	✔	✔			✔	✔	✔

**Glowacki et al 2022**	Evaluation (implementation)	Canada	✔	✔	✔			✔	✔

**Halsall et al 2020**	Description	Canada	✔	✔	✔	✔	✔	✔	✔

**Henderson et al 2022**	Evaluation (implementation)	Canada	✔	✔	✔	✔	✔	✔	✔

**Hetrick et al 2017**	Literature review	Australia	✔	✔	✔			✔	✔

**Illback et al 2010**	Evaluation (implementation)	Ireland	✔	✔	✔	✔	✔	✔	✔

**Malla et al 2018**	Evaluation (impact & implementation)	Canada	✔	✔	✔	✔	✔	✔	✔

**Mathias et al 2021**	Evaluation (implementation)	Canada	✔	✔	✔	✔	✔	✔	✔

**McGorry et al 2019**	Perspective	Australia	✔	✔			✔		

**McGorry et al 2022**	Literature review	Australia	✔	✔		✔	✔	✔	

**Mufson et al 2018**	Evaluation (impact & implementation)	USA	✔	✔	✔				

**Nadeau et al 2012**	Evaluation (implementation)	Canada	✔	✔	✔			✔	✔

**O’Reilly et al 2021**	Description	Ireland	✔	✔			✔	✔	✔

**Pomare et al 2018**	Evaluation (implementation)	Australia		✔	✔				

**Rapp et al 2017**	Evaluation (implementation)	USA	✔	✔	✔				

**Richardson et al 2014**	Evaluation (impact)	USA	✔	✔	✔				

**Rickwood et al 2020**	Description	Australia	✔	✔	✔	✔	✔	✔	✔

**Salmon et al 2021**	Evaluation (implementation)	Canada	✔	✔	✔	✔	✔	✔	✔

**Schlesinger et al 2022**	Literature review	USA	✔	✔	✔		✔		

**Scott et al 2009**	Description	Australia	✔	✔	✔		✔	✔	

**Settipani et al 2019**	Literature review	Canada	✔	✔	✔	✔	✔	✔	✔

**Shippee et al 2018**	Evaluation (impact)	USA	✔	✔	✔				

**Weersing et al 2017**	Evaluation (impact)	USA	✔	✔					

**Wright et al 2016**	Evaluation (implementation)	USA	✔	✔			✔		

**Yonek et al 2020**	Literature review	USA	✔	✔	✔			✔	


SD = Service delivery, W = Workforce, IS&C = Information systems and communication, P&T = Products and technology, F = Finance, V = Values, LG&P = Leadership, governance, and policy.

### Service delivery

Service delivery was largely framed around the principles of continuity of care between screening, primary and mental health treatment services. These models involved avoiding unnecessary transitions of care particularly during key age or developmental transitions (i.e., childhood to adult services) [1,18]. Care coordinators were used to facilitate greater continuity of care between services, improve access to outside referrals, often via phone, help problem solve barriers as they arose, and provide extended handovers to external services. In their description of a multidisciplinary YMH model, Scott and colleagues showed that a broader range of services provided at their YMH Clinic did not compromise the number of services provided and enabled greater provision of primary care services and greater care coordination [19]. Some integrated models were also able to provide crisis support 24 hours, 7 days a week [1]. However, many integrated YMH models continued to require outside referral in the case of crisis [18,20]. The intensity of care coordination, in terms of frequency and duration over the course of the intervention varied. Weekly contact was most common, with few studies describing either twice weekly contact [21], or fortnightly contact.

Another feature of service delivery for integrated YMH was **comprehensive assessment**. The use of standardised screening tools was more commonly described in services that integrated primary care with specialist YMH care. This was particularly the case in the USA, where models used brief screening measures, for example the Patient Health Questionnaire-2, to determine which YP should receive in-depth mental health assessment by a mental health clinician [21,22]. Shippee and colleagues (2018) described use of the Patient Health Questionnaire-9 A measure to screen adolescents aged 12–17 years old at ‘well-child’ health visits and refer for further MH assessment when indicated [23]. Integrated YMH services with soft entry and self-referral tended not to use standardised screening measures to determine entry to care as they aimed to provide services to all YP who were looking for help. Rather, screening was often used to determine what kind of service and the intensity of that service as part of triage and review.

### Health Workforce

Integrated *health workforces* involved internal multidisciplinary workforce of providers working together who are trained and skilled in different professions and partnerships with external organisations, such as other mental health services, primary care, alcohol and other drug services, research entities and other sectors like education or justice. The composition and functionality of integrated workforces varied. These compositions were sometimes determined by governing bodies; this was the case with the original headspace model, which stipulated that alcohol and other drug, physical health and vocational assistance services should be provided in addition to mental health services [24]. However, many integrated YMH models also allowed for a degree of autonomy and flexibility in how multidisciplinary care was implemented at a local or regional level, as population need and workforce capacity varied significantly between sites [25]. In Australia, the UK and Ireland, integrated YMH services, were designed to include psychologists, social workers, youth workers, alcohol and drug counsellors, general practitioners and psychiatrists [24]. While not necessarily mutually exclusive, some highly integrated services incorporated team members that had blended roles. For example, in the US, team-based care commonly included a primary care physician, and either a social worker, psychologist, or mental health nurse, and a psychiatrist in a consultation role [26]. As such, individual clinicians were involved with delivering multiple components of the intervention as required by the individual client [27].

### Health Information Systems and Communication

Shared communication was described as the cornerstone of most integrated interventions, with shared treatment plans and regular communication, either formally through team meetings or informally, in-person or via phone or electronic medical record. Pomare and colleagues evaluation of collaboration within multidisciplinary headspace centres noted that, with time, communication and collaboration can encourage the creation of local rules to overcome local uncertainties and other complex issues in a context-specific way [28]. In highly integrated services, communication and information systems were integrated through a shared electronic medical record. This allowed clinicians to easily refer and receive timely feedback on assessments and treatment plans from other team members [29]. Co-location appeared to facilitate better communication between services via increased opportunity for in-person communication between clinicians and ‘warm hand-offs’ [23]. Some models used a shared clinical register to track individual outcomes and facilitated follow up post-treatment. Clinical registries were often managed by care coordinators and were sometimes integrated with electronic health records or online platforms to help centralise patient data [21,23]. In Canada, the Frayme platform (frayme.ca) is an online platform that connects organisations with the aim of bolstering future implementation of integrated YMH services by providing clarity regarding the core components of integration [30]. This platform allows different providers to learn best practice tips to implement integrated care.

### Products and technology

Products and technology related to shared resources between services, including guidelines, training, infrastructure, and formal procedures. Some models have been able to enhance their integration by incorporating virtual tools and resources to improve access to care or to enhance collaborations [25,30,31]. headspace have developed eheadspace: a national online and telephone support service for YP aged 12–25 years and their families or friends [32]. eheadspace provides email, web chat and phone support staffed by qualified YMH professionals to improve access for YP who are known to be less likely to use traditional services. While some models highlight their use of information technology to enhance access, particularly as a comparison to face-to-face models of care, there was little mention of technology used to facilitate integration. This is a potential gap in integrated YMH models that might be addressed using technology enabled communities of practice.

### Health Financing

Integrated models require money, infrastructure, time and skills to be coordinated and balanced across different services. Funding models and regimes have been cited as a key barrier to service integration in mental health largely related to the erratic and piecemeal nature of mental health funding [8,20]. This landscape forced agencies to compete for a shrinking pool of funding, a disincentive for collaboration and transparency. To overcome this, joint planning and funding are crucial alongside adequate investment in shared infrastructure, including information and communication technology, developing workforce capacity, and investing in youth-friendly premises. For example, shared financial infrastructure can be used to further integrate mental health care. Exemplary models like Foundry and Forward Thinking Birmingham have been specially funded by a combination of local government and philanthropic foundations, with funding for the model as a whole, rather than piecemeal funding coming from different disciplines governing bodies, as is the case with many USA models [25,33]. An ongoing important question faced by stakeholders in the integration landscape is the sustainability of these models, along with future funding structures [34]. De Voursney and Huang note that financial arrangements for an integrated health system are less likely to be immediately attractive to funders because, ‘unlike successful management of chronic physical diseases, addressing mental disorders and chronic conditions…, is not likely to decrease or control service use in the near term’ [12]. However, they note, savings are possible through the prevention of inpatient placements resulting from behavioural health problems. In the Australian context, attempts to update existing funding models, particularly the additional access to psychology sessions through the ‘Better Access’ scheme on the Medicare Benefits Schedule (MBS) have failed to bridge socioeconomic and geographical disparities and has led to greater service fragmentation [35].

### Leadership, governance and policy

Leadership within the integrated care space involved a commitment of managers, to a clear, common agenda communicated across different services, with a focus on shared outcomes and deliverables. Successful integrated care, as described in the literature, required a common agenda among service partners at the management level, which needs to be clearly laid out in the planning stage and enshrined in mission statements and memorandums [1,18,31]. Staff working within the integrated YMH services develop an understanding of a common agenda through training and education during the implementation phase. Authors stated that a common agenda needed to be aligned with the goals of mental health services as a whole, e.g. recovery orientated principles [36] or the values of person-centred care [37]. For YMH services such as Foundry, the common agenda related to improving access and engagement with care, as well as early intervention and prevention of transition to more severe forms of mental illness [25]. Other aspects of a common agenda in integrated YMH services included a focus on vocational support, prioritising youth-friendly design, and promoting family engagement. Community engagement was also a goal of many integrated YMH service models, with the aim of promoting awareness of mental health needs of YP, encouraging access of services, and destigmatising mental health care [31,38]. Joint planning and commissioning of mental health services for health funders is a crucial element of integrated YMH service models. This involved partnerships between different health payers and service providers, including different levels of government (eg Federal, State or regional), as well as non-governmental organisations, and private organisations including health insurers (particularly in the US). In Australia, McGorry and colleagues describe the importance of strong national oversight of services to assure integrative commissioning and sustainable financial models [39]. Formal partnerships are also a vital part of integrated care. These may be preceded by informal alliances and networks, which may also include community groups, academia, and research partners, in addition to the stakeholders listed above. Alliances between different organisations can help establish a common vision and goals and can lead to working groups and steering committees essential to service development. Despite this, ‘distributive leadership’, which is “an approach involving concerted action achieved by spontaneous collaboration through intuitive working relationships” [40: p1], was found to be a facilitator of service and system-level integration. This type of leadership was also effective in coordinating efforts for achieving optimised access to care [40].

### Values

Different approaches in people-centred and integrated health service delivery “should be grounded in a common set of principles. These provide a unifying values framework” [41: p11]. Identification of the underlying values of integrated care enables better understanding of collaboration and behaviour in integrated care and could also help to define care quality. Shared values across professionals and organisations are important factors in informal coordination and collaboration processes as noted in the Rainbow Model of Integrated Care [42]. This model is a helpful framework for considering integration with regards to micro (clinical integration), meso (professional and organisational integration) and macro (system integration) levels of a system.

### Creation of the Youth Integration Project (YIP) framework

Themes mapped to the WHO building blocks were then considered across intensity levels as outlined by Heath and colleagues [43] to create a novel YMH integration framework. Heath and colleagues outlined six intensity levels of integrated care. The first two levels focus on communication and fall under the categorisation ‘Coordinated Care’, which involves minimal or basic collaboration at a distance. The second two levels focus on geographical proximity and fall under ‘Co-located Care’, which involves on-site collaboration, and at level four some degree of system integration. The final two levels focus on practice change and are categorised under ‘Integrated Care’, which involves close/full collaboration leading to a completely transformed integrated practice. Therefore, many health professionals and researchers consider lower levels (i.e., coordinated and collocated care) to be forms of integrated care, and view ‘fully integrated care’ as the final point along a continuum or health transformation journey [43]. Table 2 details how each core component of integration occurs along the continuum of integration, adapted from Heath and colleague’s work [43]. Supplementary file 2 is a tool we have created to guide the identification of integration across these core components.

**Table 2:** Levels and components of Integration: The Youth Integration Project (YIP) Framework*.


	COORDINATED	CO-LOCATED	INTEGRATED

**Service delivery**	Separate screening, treatment plans, and evidence based practices.	Agree on specific screening. Separate service plans informed by some shared knowledge. Some shared EBPs and training	Consistent screenings across disciplines. Shared treatment planning. EBPs and training shared across system

**Health Workforce**	Multidisciplinary workforce. No appreciation of each other’s culture. View each other as outside resources	Multidisciplinary workforce. Some appreciation of each other’s role. One discipline overshadows others.	Multidisciplinary workforce. In-depth appreciation of roles and culture. Shared sense of ownership of model

**Information Systems and Communication/Products and technology**	Separate facilities. Separate systems. Communicate rarely	Co-location. Separate systems. Communicate occasionally	Co-location. Shared systems. Face-to-Face consultation. Regular formal and informal meetings and communication.

**Finance**	Separate funding. Limited sharing of resources. Separate billing practices	Separate funding but may share grants. Some sharing of costs. Separate billing due to system barriers	Integrated funding from multiple sources of revenue. Resources shared and allocated. Billing maximised for integrated model and single billing structure

**Leadership, governance, and policy/ Values**	No shared vision. Limited shared leadership. Limited provider buy-into collaboration.	Some shared vision. Organisation leaders support integration nominally. Some buy-in to integration but not consistent across all providers.	Documented shared vision clearly communicated. Organisation leaders strongly support integration. Integrated care and all components embraced by providers


*A more complete version of the table is included as supplementary file 3.

### Applying the framework

The YIP framework is intended to be used to evaluate the level of integrated care specifically in YMH. In the following examples, we demonstrate how the YIP framework can be applied to models or systems of integrated care to explore how integrated they are, and where improvements in integration can occur across health system building blocks. We present two models from our literature review as contrasting case examples demonstrating different levels and aspects of integration (Presented in Tables 3, 4, 5).

**Table 3:** headspace integration evaluation.


BUILDING BLOCKS	INTEGRATION EVALUATION AS PER THE YIP FRAMEWORK (TAKEN FROM (28))

**Service Delivery**	A single, visible location, a on stop shop with providers providing the full spectrum of care around a young person and his/her family. Focus on early intervention approach offering safe, holistic, evidence-informed, proportional and stage-linked care, including risk-benefit considerations and shared decision-making, with social and vocational outcomes as the key targets. **(Level 5/6)**.

**Health Workforce**	Centres are staffed by multidisciplinary teams comprising mental health, physical health, alcohol and other drug, and vocational support along with non-clinical (peer worker) staff. Workforce capacity is a challenge for some centres, particularly those in rural and remote locations where a full complement of the necessary workforce may not be available. **(Level 5/6)**.

**Health Information Systems and Communication/Products and Technology**	On-site integration is achieved within the headspace centre and co-located services through collaborative care planning and delivery, shared-care arrangements and multidisciplinary case review. headspace centres are required to maintain an up-to-date register of other services in the community that YP might need. Strong partnerships, established referral pathways and warm referrals are used to integrate care with external service providers. **(Level 5/6)**.

**Leadership, Governance, and Policy/Values**	headspace has a foundation in touth (and family) participation and co-design at all levels. National network oversight is balanced against local context-specific governance. headspace centres model are governed by a Lead Agency i.e. an independent organisations commissioned to operate each headspace centre. The Lead Agency provides the infrastructure and is responsible for corporate and clinical governance. Additional governance is provided by a Consortium of local service providers that collaborate with the Lead Agency to give strategic direction, additional capacity through in-kind contributions and local planning oversight. **(Level 5/6)**.

**Funding**	Multiple funding streams are combined to support a headspace centre. The Australian Government Department of Health provides core funding which covers infrastructure and salaries for essential staff positions. The Australian Government’s Medical Benefits Scheme rebates medical and allied health staff for designated health services. In-kind contributions are expected from Consortium member and local partner organisations to provide the full range of services. Additional state/territory government funding is provided to some centres. Core staff are directly employed through the headspace centre grant, while others are employed through contracted private practitioner arrangements or via in-kind contributions. **(Level 5/6)**.

**Overall Level**	**Level 5/6 Integrated as per the YIP Framework**


**Table 4:** Foundry integration evaluation.


BUILDING BLOCKS	INTEGRATION EVALUATION AS PER THE YIP FRAMEWORK (TAKEN FROM (22))

**Service Delivery**	Diverse services co-located and accessed individually or concurrently, and staff and organizations work collaboratively so that YP experience seamless care, in a single visit, many youths access one or more of Foundry’s five distinct services (i.e., primary care, mental health care, substance use support, peer support, and/or social services). **(Level 5/6)**.

**Health Workforce**	Services at each centre include primary care (physical and sexual health), mental health, substance use, peer support and social services (e.g., employment, housing, and income assistance) **(Level 5/6)**.

**Health Information Systems and Communication/Products and Technology**	Same Facilities; Shared systems; Face-to-Face consultation; Have formal and informal meetings to support integrated model of care. Foundry Virtual (foundrybc.ca) offers YP and their caregivers drop-in counselling, peer support, primary care, and youth relevant information and resources online. **(Level 5/6)**.

**Leadership, Governance, and Policy/Values**	Foundry’s leadership structure, comprising a provincial Governing Council, Foundry Central Office, and Lead Agencies (LA) support the development of Foundry centres through integrating services and practices within a complex system. The Foundry central office leads the provincial initiative and supports the development of local centres. Each Foundry centre is operated by a lead agency that brings together local stakeholders. **(Level 5/6)**.

**Funding**	Lead Agencies were selected in each community to have organisational accountability for the overall financial management and service delivery accountability for their centre. However, by agreement with all partners, Lead Agencies rely heavily on direct and indirect contributions from partnering agencies to deliver all onsite services, thus requiring a coordinated and collaborative approach. **(Level 5/6)**.

**Overall Level**	**Level 5/6 Integrated as per the YIP Framework**


**Table 5:** SCIPT-A integration evaluation.


BUILDING BLOCKS	INTEGRATION EVALUATION AS PER THE YIP FRAMEWORK (TAKEN FROM (43))

**Service Delivery**	Combined screening stepped care model, EBP: SCIPT-A (phase I: 8 weeks of weekly IPT, phase II: 8 weeks of either weekly sessions, or 3 sessions in total) implemented by social worker. Pharmacotherapy implemented by PCP. **(Level 1/2)**.

**Health Workforce**	Clinic social worker (master’s level), PCP (7 paediatricians and 1 nurse practitioner). Trained separately. **(Level 1/2)**.

**Health Information Systems and Communication/Products and Technology**	Same Facilities. No detail about systems. Clinic social worker and PCP would collaborate after an assessment of patient’s response to treatment and synthesis. “… medical providers reported the need for improved communication with social work clinicians and back-up support with a consulting psychiatrist to implement the model successfully” **(Level 1/2)**.

**Leadership, Governance, and Policy/Values**	Mental health focused intervention. Siloed delivery of service. Limited data on shared vision **(Level 3)**.

**Funding**	Funded by research grant (National Institute of Mental Health Grant) therefore no funding buy in from local stakeholders **(Level 1–4)**.

**Overall Level**	**Level 1/2 Coordinated as per the YIP Framework**


#### headspace (Table 3)

headspace is a foundational integrated YMH model using the headspace centre as an easy-access, youth-friendly, integrated primary care service that partners with services in the local community to provide an early intervention approach to mental health problems for YP aged 12 to 25 years. The headspace model is based on removing the barriers to service access and increasing the propensity for YP to seek help at this stage of life [31]. Recently, centres have been strengthened in six regions by vertical integration with specialized services for more complex, low prevalence disorders, notably early presentations of psychosis. Further, the national headspace initiative provides other services and programs including an online YMH service “eheadspace”, headspace mental health in education services, the headspace interactive website, and a digital work and study service, among others.

#### Foundry (Table 4)

Foundry is a province-wide network of integrated health services designed for YP aged 12–24 years in British Columbia, Canada, located in both urban and rural communities. Beginning in 2015 with six centres, it has since grown to 11 centres, with another eight centres due to be operational by 2023. Foundry services include primary care (physical and sexual health), mental health, substance use, youth and family/caregiver peer support and social services (for employment, housing, income support), all provided under one roof. In the period of April 2018 to September 2020 Foundry provided over 100,000 occasions of service to YP [44]. The Canadian integrated YMH model Foundry consists of partnerships with over 200 government and non-profit community-based organisations [25,44]. Foundry was initially conceived as a “collective impact” initiative, with the Foundry Central Office acting as its “backbone” organisation. Foundry has engaged over 140 partners across the province of British Columbia. Centres are governed by lead agencies and guided and supported by Foundry Central Office and a provincial Governing Council [40]. While in the proof-of-concept phase, none of the centres achieved “target” results for any of the constructs measured, which related to partnership functioning (for example synergy, administrative and management effectiveness, sufficiency of resources), several were categorised as making ‘headway’ [45].

#### SCIPT-A: Stepped collaborative care treatment model (Table 5)

Mufson and colleagues examined the feasibility and acceptability of a stepped collaborative care model involving clinic social worker delivered interpersonal psychotherapy for depressed adolescents (IPT-A) to an underrepresented minority sample with the option, when needed, to intensify treatment by adding medication delivered by the primary care provider compared to Enhanced Treatment as Usual (E-TAU) [46]. Based in the USA, this model, SCRIPT-A, was delivered in a paediatric clinic by clinic staff. Participants randomized to SCIPT-A received 8 weekly sessions of brief IPT-A in Phase I, followed by either maintenance treatment (if the adolescent responded) or combined IPT-A plus medication (if the adolescent did not respond sufficiently) in Phase II. The model included opportunities for the clinic social worker and paediatrician to collaborate and synthesise the clinical data to determine the next steps needed to help achieve recovery.

## Discussion

Our review of the literature on integrated YMH care outlined the current definitions of integrated YMH, including levels of integration. The review also consolidated the literature on the core components of integrated care, further defining service delivery; workforce; information systems and communication; products and technology; leadership governance and policy; finance; and values as key features of integrated care in YMH. We have used this review to develop a novel framework, the YIP framework, which can be applied to evaluate and strengthen integration in YMH.

To date, there has been definitional and conceptual confusion around what integrated care involves [47]. Our description of the core components of integrated care aims to help address this gap in the literature. Further, the review expands and enhances Yonek and colleagues’ recent narrative review of the key components of effective paediatric integrated mental health care models [48]. We noted that many of the components included in Yonek and colleagues’ summary were not specifically system level features of integrated care, but rather reflected good clinical practice in YMH generally. For example, the inclusion of population-based care, brief psychological intervention, and medication therapy as key components in Yonek and colleagues’ review do not relate to how services collaborate to provide care. Our findings differ due to our focus on the systems level features of models related to integration rather than clinical intervention features. Our framework can evaluate health systems and models and identify ways to strengthen integration across core components.

Of particular importance within our framework is service delivery. Within YMH, service delivery is particularly fragmented in terms of age (paediatric to adult), disorder (e.g. psychosis, personality disorder, drug and alcohol), setting (primary, emergency department, crisis level, specialist community mental health, non-government organisations (NGO)), and discipline (e.g. psychiatric, primary care, clinical psychology, social work). This fragmentation leads to duplication and potentially unnecessary transitions in care, wasting resources and increasing the risk of consumer disengagement. Service delivery fragmentation poses challenges for YP accessing and navigating different services and limits opportunities for cross service collaboration. Our framework maps out the material trajectories of service delivery in terms of a patients experience within the system through screening and assessment, referral processes, delivery of services, and ceasing care. The other elements of the framework, from workforce to values can be considered in terms of how they are organised to leverage and support care delivery at the coalface.

Previous work has developed frameworks to consider integration such as the International Foundation for Integrated Care’s nine pillars of integrated care, which includes: shared values and vision, population health and local context, people as partners in care, resilient communities and new alliances, workforce capacity and capability, system wide governance and leadership, digital solutions, aligned payment systems, and transparency of progress, results, and impact [49]. The YIP framework’s novel contribution is the ability to apply it to evaluate health systems and models and identify ways to strengthen integration across core components. The Rainbow Model of Integrated Care (RMIC) [42] also helped us to particularly consider the specific shared values across integration contexts. Work similar to our own has recently been published using the RMIC to help determine whether integrated hub models of care improve mental health outcomes for children experiencing adversity [50]. In their paper, Honisett and colleagues use the RMIC to evaluate levels of integration among studies included in their review. However, we believe that the RMIC poses difficulties for clinicians and academics implementing integrated models of care; specifically, the separation of factors of integration across micro, meso, and macro contexts. Distinctions across these planes of implementation in real world contexts are rarely distinct and discrete. The YIP framework implies these distinctions (for example, the components of policy, governance, and finance are more likely to sit within a macro context) without having to consider them in isolation.

While emerging evidence for integrated care models to meet the current needs of the YMH system appears promising [20,51], more work is needed to determine whether comprehensive integrated, co-located services for mental and physical (including sexual) health, substance use, vocational and social support are a viable and effective way to deliver services. There remains a paucity of information related to the impact, implementation, and cost effectiveness, of these kinds of models. There is also limited evidence to guide the maintenance and sustainability of these models, along with future funding structures [34,52]. Additionally, the changing nature of service delivery predicated on the role of technology in response to the COVID-19 pandemic may provide opportunities and challenges for integration that need to be considered. Technology can facilitate integration but cannot be considered a panacea ignoring other core components of integration.

A limitation of our review is the narrow focus in scope on models of care where mental health is a substantial component. We acknowledge the importance of models which provide integrated health and social care to YP with complex needs, for instance the New Zealand Youth One Stop Shops [53]. Considering how our theoretical framework might apply to integration between mental health systems and physical health systems is an important next step for future work. Another limitation of our review is the challenges around examining some components of integration within published models, which were often poorly described within the literature. For example, the design of governance structures, the development of intersectoral partnerships and shared funding were not forthcoming across many papers included within this review. Relatedly, our evaluation of integrated models of care using our framework is limited to the veracity of the details provided in publications describing the models. A key recommendation from this review is greater transparency of system level levers of integrations within YMH research on integrate care.

## Conclusion

Real world experience of how YMH integration can be delivered at a service level, through structural and systemic redesign, is needed to support and inform local integration efforts, ensuring YP can access appropriate mental health care. The YIP framework can help support the development and evaluation of integrated YMH pathways. Specifically, the framework can map the barriers and enablers to YMH integration; support testing co-produced and evidence-based solutions to address service fragmentation; and help inform policy development.

## Additional Files

The additional files for this article can be found as follows:

10.5334/ijic.7730.s1 Supplementary File 1. Search terms.

10.5334/ijic.7730.s2 Supplementary File 2. Tool for guiding and evaluating service integration.

10.5334/ijic.7730.s3 Supplementary File 3. The Youth Integration Project (YIP) Framework unabridged.
