# Supplementary material for: Creation of the Youth Integration Project Framework: A Narrative Synthesis of the Youth Mental Health Integrated Care Literature
Source: Int J Integr Care. 2024 Jul 5;24(3):5. doi: 10.5334/ijic.7730 (PMC11225559; doi:10.5334/ijic.7730)
Supplement: Supplementary File 1. — Search terms. [file ijic-24-3-7730-s1.pdf]

Supplementary file 1: Search terms

Paediatric group:

pediatric* OR paediatric* OR teen* OR adolescen* OR pubescent OR “young people” OR youth* OR pubert* OR “young adult”.

Mental health condition:

“mental health*” OR “mental disorders” OR “mental health services*”.

Integrated care model:

“integrat*” OR “integrated care” OR “colocat*” OR “care coordination” OR “collaborative care” OR “coordinated care” OR “horizontal integration” OR “vertical integration” OR “longitudinal integration” OR “virtual integration” OR “medical home” OR "health services*" OR "delivery of health care*".
