# Supplementary material for: Creation of the Youth Integration Project Framework: A Narrative Synthesis of the Youth Mental Health Integrated Care Literature
Source: Int J Integr Care. 2024 Jul 5;24(3):5. doi: 10.5334/ijic.7730 (PMC11225559; doi:10.5334/ijic.7730)
Supplement: Supplementary File 2. — Tool for guiding and evaluating service integration. [file ijic-24-3-7730-s2.pdf]

Tool for guiding and evaluating service integration

Core Component	Measure	Data	
**Service Delivery**	Describe screening process		
	Describe referral pathways	**[Attach referral pathways summary document]**	
	List shared evidence-based practices i.e. guidelines		
**Health workforce**	List the services co-located withing the model.		
	Indicate days per week staffed be service 1. (Please circle)	**Monday Tuesday Wednesday Thursday Friday**	
	Indicate days per week staffed be service 2. (Please circle)*	**Monday Tuesday Wednesday Thursday Friday**	
	Indicate links with other external services (if any).		
**Information systems and Communication**	Identify electronic medical records used by services	**Service**	**EMR**
	List joint meetings between disciplines/services	**Meeting**	**Frequency**

**Products and technology**	List any shared resources between services e.g. *Infrastructure (buildings, services i.e. security, administration), communication technology, digital resources (i.e. Facebook page)*		
	List any joint training shared between services e.g. *Common Agenda training*		
**Finance**	List the funding source of each of the services included in the model	**<u>Service</u>**	**<u>Source of funding</u>**
**Leadership, governance, and policy**	List any joint strategy activities undertaken between services *e.g. planning days, strategic plan*		
	**[Attach document indicating the model governance structure]**		
	Provide the documented shared vision (if any).		
**Values**	Identify the shared values of the services involved in the model.		

*Add more as necessary
