# Supplementary material for: Creation of the Youth Integration Project Framework: A Narrative Synthesis of the Youth Mental Health Integrated Care Literature
Source: Int J Integr Care. 2024 Jul 5;24(3):5. doi: 10.5334/ijic.7730 (PMC11225559; doi:10.5334/ijic.7730)
Supplement: Supplementary File 3. — The Youth Integration Project (YIP) Framework unabridged. [file ijic-24-3-7730-s3.pdf]

Supplementary file 3: The Youth Integration Project (YIP) Framework unabridged

	Coordinated<br>Key Element: Communication		Co-Located<br>Key Element: Physical Proximity		Integrated<br>Key Element: Practice Change	
	LEVEL 1<br>Minimal Collaboration	LEVEL 2<br>Basic Collaboration at a distance	LEVEL 3<br>Basic Collaboration Onsite	LEVEL 4<br>Close Collaboration Onsite with Some System Integration	LEVEL 5<br>Close Collaboration Approaching an Integrated Practice	LEVEL 6<br>Full Collaboration in a Transformed/ Merged Integrated Practice
**Service delivery**	• Screening based on separate practices<br>• Separate treatment plans<br>• Evidenced-based practices (EBP) implemented separately	• Screening based on separate practices<br>• Separate treatment plans (may be shared)<br>• Separate responsibility for care/EBPs	• Agree on specific screening<br>• Separate service plans informed by some shared information<br>• Some knowledge of each other's EBPs, especially for high utilizers	• Agree on specific screening<br>• Collaborative treatment planning for specific patients<br>• Some EBPs and some training shared, focused on interest or specific population needs	• Consistent set of agreed upon screenings across disciplines<br>• Collaborative treatment planning for all shared patients<br>• EBPs shared across system with some joint monitoring of health conditions for some patients	• Population -based screening is standard practice with results available to all<br>• One treatment plan for all patients<br>• EBPs are team selected, trained and implemented across disciplines as standard practice
**Health Workforce**	• Multidisciplinary workforce<br>• Little to no appreciation of each other's culture	• Multidisciplinary workforce<br>• Little understanding of each other's culture or sharing of influence<br>• View each other as outside resources	• Multidisciplinary workforce;<br>• Some appreciation of each other's role and general sense of large picture<br>• Mental health usually has more influence	• Multidisciplinary workforce<br>• Basic appreciation of each other's role and cultures	• Multidisciplinary workforce<br>• In-depth appreciation of roles and culture	• Multidisciplinary workforce<br>• In-depth appreciation of roles and culture

**Information Systems and Communication /Products and technology**	• Separate facilities<br>• Separate systems<br>• Communicate rarely (Only in emergency or uncommon circumstances)	• Separate facilitates<br>• Separate systems<br>• Periodic focused communication about shared patients; mostly written)	• Same Facilities<br>• Separate systems<br>• Communicate regularly about shared patients, by phone or e-mail	• Same Facilities<br>• Separate systems<br>• Communicate in person as needed<br>• Joint consultation<br>• Coordinated reatment plans	• Same Facilities<br>• Shared systems<br>• Face-to-Face consultation<br>• Have regular team meetings to discuss overall patient care and specific patient issues	• Same Facilities<br>• Shared systems<br>• Face-to-Face<br>• consultation<br>• Have formal and informal meetings to support integrated model of care
**Finance**	• Separate funding<br>• No sharing of resources<br>• Separate billing practices	• Separate funding<br>• May share resources for single projects<br>• Separate billing practices	• Separate funding<br>• May share facility<br>• Expenses<br>• Separate billing practices	• Separate funding, but may share grants<br>• May share office<br>• expenses, staffing costs, or infrastructure;<br>• Separate billing due to system barriers	• Blended funding based on contracts, grants or agreements<br>• Variety of way to structure the sharing of all expenses<br>• Billing function combined or agreed upon process	• Integrated funding based on multiple sources of revenue<br>• Resources shared and allocated<br>• Billing maximised for integrated model and single billing structure
**Leadership, governance, and policy/ Values**	• No shared vision<br>• No coordination or management of collaborative efforts<br>• Little provider buy-in to integration or even collaboration	• No shared vision<br>• Some practice leadership in more systematic information sharing<br>• Some provider buy-into collaboration	• Some shared vision<br>• Organisation leaders supportive but often colocation is viewed as a project or program<br>• Provider buy-in to making referrals work and appreciation of onsite availability	• Some shared vision;<br>• Organisation leaders support integration through mutual problem-solving of some system barriers<br>• More buy-in to concept of integration but not consistent across all providers.	• Documented shared vision clearly communicated<br>• Organisation leaders support integration if barriers minimal<br>• Nearly all providers engaged in integration model	• Documented shared vision clearly communicated<br>• Organisation leaders strongly support integration as practice model with expected change in service delivery<br>• Integrated care and all components embraced by providers
